# Early postoperative complications in patients with Crohn's disease given and not given preoperative total parenteral nutrition

**DOI:** 10.3109/00365521.2011.648954

**Published:** 2012-01-12

**Authors:** Stefan Jacobson

**Affiliations:** The Department of Surgery, Karolinska Institute, Huddinge Hospital, Stockholm, Sweden

**Keywords:** Crohn's disease, parenteral nutrition, postoperative complications

## Abstract

**Objective:**

The effect of preoperative total parenteral nutrition (TPN) on the rate of early (within 30 days) postoperative complications in patients with moderate to severe Crohn's disease (CD) was examined.

**Material and methods:**

A series of 15 consecutive patients with CD (mean CD activity index score, 270) given preoperative TPN for 18–90 days (mean, 46 days) and undergoing bowel resection and primary anastomosis was compared with matching controls (105 patients) consecutively selected from all CD patients operated in Stockholm County during a preceding 20-year period without preoperative TPN.

**Results:**

During the preoperative TPN, all the patients studied displayed clinical remission of CD as reflected in improvement in their general well-being, relief of abdominal pain, and abatement of fever and diarrhea. There was no significant early postoperative complication in the TPN-treated group, whereas there were 29 patients with early postoperative complications in the control group, which means a significantly higher rate of postoperative complications when preoperative TPN was not provided. During the preoperative TPN, some crucial variables increased such as the body weight, the serum concentrations of albumin and triiodothyronine reflecting improved nutritional state, whereas the serum concentration of haptoglobin and the white cell count decreased reflecting decreased inflammatory activity.

**Conclusions:**

This study shows that preoperative TPN for at least 18 days may be recommended to be given to patients with moderate to severe CD until clinical remission is achieved in order to minimize the risk of early postoperative complications.

## Introduction

In 1932, Crohn's disease (CD) was clearly described as an entity by B. B. Crohn and colleagues, who reported 14 patients who had been operated by Dr. A. A. Berg [1]. Accumulating evidence suggests that CD results from an inappropriate response to intestinal microbes in a genetically susceptible host [2]. Since about 70% of all patients with CD ultimately come to surgery [3], and of all patients undergoing first resections for CD nearly 50% will ultimately require a second operation [4], there has been considerable interest in the outcome of surgical treatment for this condition.

The results of surgical treatment of CD at the Mount Sinai Hospital, where B. B. Crohn worked, were reviewed in 1945, when the overall mortality for primary resection was over 13%, and in 1985 when there was no mortality in a series of 130 patients [5]. However, 30% of these 130 patients studied developed postoperative complications, which were classified as major in 8% [5].

Emergency surgery is seldom necessary in CD. Thus, spontaneous free perforation and perforated abscess were found to occur in only 2.1% in a series of 1415 CD cases [6].

Whereas malnutrition has been associated with increased postoperative mortality and morbidity rates since 1936 [7,8], convincing evidence of significant reduction of postoperative complications by preoperative total parenteral nutrition (TPN) is rare and sometimes controversial [9,10].

In the present study, a series of CD patients given preoperative TPN was compared with a series of matching CD patients who had not been given preoperative TPN. If a relationship between preoperative TPN and absence of postoperative complications exists, a significantly higher rate of postoperative complications would be expected to be present in the series of CD patients, who had not received preoperative TPN.

## Patients, materials, and methods

### Cases

The cases consisted of a series of 15 consecutive CD patients given preoperative TPN (nil per os) during 18–90 days (mean, 46 days) followed by intestinal resection with primary handsewn anastomosis end-to-end usually performed with a single-layer of interrupted absorbable synthetic suture material (Dexon) (group 1). In one patient, only the sigmoid colon was resected, in another one the intestine on both sides of a jejunocolonic anastomosis was resected, whereas in the other 13 patients an ileocolonic resection was performed. All patients were given infection prophylaxis with antibiotics postoperatively [11]. The preoperative TPN was given on medical grounds while the patients were hospitalized. The diagnosis of CD was based on the findings at histologic examination [12]. During the period studied, no CD patient in a serious condition was refused preoperative TPN. CD patients with only symptoms of intestinal stenosis and no sign of malnutrition and no particular sign of inflammation were not included in the study. All patients gave their informed consent prior to their inclusion in the study, which was performed in accordance with the ethical standards laid down in the 1975 Declaration of Helsinki.

The clinical data of the patients in group 1 are presented in Tables I and II. Intestinal resection had previously been performed in six patients. At the introduction of TPN, three patients were given prednisolone, 10–40 mg/24 h, but none received or had received treatment by means of biologicals.

**Table I:** Clinical data for the patients in group 1 at introduction of total parenteral nutrition.

	Number	Sex	Age (yr)		Height (cm)		Body weight (kg)		BMI^a^ (units)		CDAI^b^ (units)	
		M/F	Mean	SD	Mean	SD	Mean	SD	Mean	SD	Mean	SD

Group 1	15	4/11	35	12	172	9	54	9	18.6	1.8	270	107

a BMI = body mass index = weight/(height)^2^;

b CDAI = Crohn's disease activity index [40].

**Table II:** Disease pattern and previous operations for Crohn's disease (CD) in the patients of group 1 when total parenteral nutrition (TPN) was introduced.

Disease pattern	Group 1 *n* = 15
Ileitis	8^a^
Ileocolitis	5^b^
Colitis	2^c^

a One patient had undergone exploratory laparotomy 1.5 months earlier. Another patient had undergone ileocecal resection 4 years earlier. A third patient had undergone appendectomy and ileotransversostomy 7 years and 1 month earlier, respectively, and a fourth patient had undergone appendectomy and ileocecal resection 7 and 6.5 years earlier, respectively;

b One patient had undergone appendectomy and segmental resection of the ileum 11 and 2 years previously, respectively. Another patient had undergone colonectomy one month earlier and a third patient had undergone appendectomy, ileocecal resection, exploratory laparotomy, and two ileocolonic resections 35, 4, 3, 2, and 2 years earlier, respectively;

c One patient had undergone appendectomy, ileotransversostomy, and ileocolonic resection 22, 20, and 19 years previously, respectively.

### Controls

Age, sex, disease pattern, type of operation, and the number of previous operations for CD are factors that may all relate to the rate of postoperative complications. To control for the potential effects of these factors, a sevenfold multiple of controls (the highest obtainable multiple) were matched to the 15 cases on the basis of the above-mentioned factors. The controls were consecutively selected from all the 692 CD patients operated without preoperative TPN at the 14 acute hospitals in Stockholm County during a 20-year period ending a few years before the beginning of the present series of 15 CD cases studied [13].

Matching was accomplished in three steps. First, the controls, who had undergone the same type of intestinal resection and anastomosis and had the same disease pattern and had undergone the same number of previous operations (1, 2, or 3–9) for CD as one of the 15 cases, were matched to each such case. Second, the sevenfold matching was adjusted on the basis of sex, and third, on the basis of age (within the same decade).

For one case operated with segmental resection of the colon, controls had to be selected from both sexes and from four decades to obtain seven corresponding controls. For three other but between themselves similar cases, only 20 corresponding controls were found within the limitations set; an extra control free from postoperative complication was therefore included to obtain a sevenfold matching. For yet another case, only six corresponding controls were found; again an extra complication-free control was included for the same reason. Thus, 105 controls constituting group 2 were found matching to the 15 CD cases.

Before the preoperative TPN given to group 1 and before intestinal resection in group 2, there was no difference in steroid treatment and treatment by means of biologicals between the patients in groups 1 and 2.

### Infusion solutions and procedures

In the TPN given group 1, the infusion solutions included amino acids (Vamin with glucose or fructose, Pharmacia, Stockholm, Sweden), carbohydrates, fat emulsion (Intralipid 10% or 20%, Pharmacia) [14], electrolytes, trace elements (Addamel, Pharmacia), and vitamins (Soluvit and Vitalipid Adult, Pharmacia) [15]. The TPN regimens provided 9.8–13.1 MJ (2350 to 3100 kcal) of energy per 24 h (8.8 to 11.5 non-protein MJ/24 h) in 2.5–3.6 liter/24 h including 53–106 g of fat (only two patients were given 106 g of fat per 24 h; the others received 53–56 g/24 h), 65–438 g of glucose, 113–234 g of fructose, 6–11 g of glycerol (in the fat emulsion, Intralipid 10% and 20%), 3–4.5 g of sorbitol (in the trace element solution, Addamel), 9.4–14.1 g of nitrogen, 160–195 mmol sodium, 70–110 mmol potassium, 7.5–11.3 mmol calcium, 4.1–9.5 mmol magnesium, 13.5–19.5 mmol phosphate, one ampoule of Soluvit (water-soluble vitamins), one ampoule (10 ml) of Vitalipid Adult (fat-soluble vitamins), and 10–15 ml of Addamel.

The intravenous infusions were given for about 14 h daily (08.00–22.00 h) by gravity drip through a central venous catheter (CVC). A heparin lock was placed for the remainder of the 24-h period. The duration of the preoperative TPN in group 1 was 46 ± 22 days (mean ± SD).

In the early cases of the series, all infusion components were mixed at the pharmacy. In the last two cases, the fat emulsion, including the water- and fat-soluble vitamins, was given from 08.00 to 14.00 h and the mixture of the other infusion components, delivered from the pharmacy in a 3-liter plastic bag (Travenol, Thetford, Norfolk, England), was given from 08.00 to 22.00 h; the fat emulsion and the carbohydrate-amino acid solution were mixed as they passed through the CVC. To reduce the risk of CVC-related thrombosis, five of the last patients were given heparin intravenously, 5000 IU/6 h [16].

One of the cases was given 0.5 liter of packed erythrocytes on days 11–12 of the preoperative TPN. The blood component therapy was given for anemia, which had been present at the introduction of TPN. None of the other cases was given whole blood or blood components during the preoperative TPN.

### Definition of early postoperative complications

Early postoperative complications are defined as complications within 30 days postoperatively [17]. Wound infection was defined as spontaneous discharge of pus from the wound. Perforation with free peritonitis was documented at laparotomy. A wound dehiscence required operative re-closure and intestinal obstruction required laparotomy for decompression. Enter-ocutaneous fistula was documented radiologically and by the discharge of intestinal contents through the abdominal wall. Entero-enteric fistula was revealed at laparotomy, occasionally by barium enema preoperatively. A perianal fistula was disclosed by a fistulous opening adjacent to the anus. Intraabdominal abscess and anastomotic leakage were documented at operation. Intraabdominal bleeding required laparotomy for hemostasis. Pneumonia was documented by an abnormal chest roentgenogram.

### Analytical methods

After an overnight fast (12–14 h), venous blood samples were drawn on one of the 3 days preceding TPN and at intervals during the period of TPN before the daily infusion was started. In blood (B-) reticulocytes, white cell count (WBC) and the concentration of hemoglobin (Hb) and in serum (S-) or plasma (P-) the concentrations of albumin, orosomucoid, αl-antitrypsin, haptoglobin, asparagine aminotransferase (ASAT), alanine aminotransferase (ALAT), alkaline phosphatase (ALP), total bilirubin, cholesterol, triglycerides (TG), triiodothyronine (T3), thyroxine (T4), thyrotropic hormone (TSH), and the immunoglobulins G, A and M (IgG, IgA, and IgM, respectively) were determined by the conventional methods used at Huddinge Hospital. Free fatty acids (FFA) in serum were determined by a gas chromatographic method [18]. Total phospholipids (PL) in serum were determined according to Svanborg and Svennerholm [19] and individual PL according to Vikrot [20].

Univariate comparisons of proportions were made by the *X*^2^ test with Yates's correction. The statistical significance of differences was examined by “Student's” paired and unpaired t-tests. For all tests, *p* value of less than 0.05 was considered significant. The results are reported as the mean ± standard deviation (SD).

## Results

During the preoperative TPN, all the cases in group 1 displayed clinical remission of CD – as judged from their general well-being, relief of abdominal pain, and abatement of fever and diarrhea. Although enterocutaneous and perianal fistulas healed and abdominal mass disappeared during the TPN, there were more or less stenotic intestinal CD changes remaining, which were removed at the following operation. When clinically sufficient remission had been achieved, but X-ray examination showed remaining intestinal stenosis, bowel resection was considered indicated. At operation, CDAI was estimated to be well below 150 in every TPN patient, and CDAI < 150 is considered to be in remission by this measure.

There was no significant postoperative complication in the TPN group, whereas there were 29 patients with postoperative complications in group 2 (Table III). This is a higher rate of complications (*p* < 0.05) than in group 1. During the preoperative TPN, however, there were some complications but no central line-associated blood stream infection. Thus, because of CVC-associated thrombosis usually presented as ceased infusion, one patient successively was given five CVC:s during 45 days of preoperative TPN (she had been given TPN for 2 months as the sole therapy 7–9 months earlier and then probably developed subclavian and superior caval vein thromboses, angiographically verified after the fourth CVC the last CVC was introduced via vena saphena magna), another patient needed three consecutive CVC:s during 76 days of preoperative TPN, a third needed two CVC:s during 54 days of preoperative TPN, and a fourth patient also needed two CVC:s during 47 days of preoperative TPN, whereas the remaining 11 patients had uneventful courses of preoperative TPN. When the fourth patient with CVC-associated thrombosis after 31 days of preoperative TPN was to be given the second CVC, an attempt to insert a subclavian intravenous catheter resulted in a pneumothorax cured by 5 days of Bülau drainage. At operation, all TPN patients were in good condition.

**Table III:** Distribution of postoperative complications in 29 of the 105 patients in group 2.

Complication	Number of patients
Death	1
Wound complication	14
Intestinal obstruction	6^a^
Fistula	4
Anastomotic leakage	2
Other^b^	2

Total	29

a One patient had moreover anastomotic leakage;

b One had blood transfusion hepatitis and another patient suffered a very complicated postoperative course including enterocutaneous fistula, abscess in the rectouterine pouch and cachexia.

All patients in group 1 were discharged from Huddinge Hospital within 30 days postoperatively (17 ± 7 days); the last eight cases were discharged after only 12 ± 4 days. Because of relatively low Hb values on the day of operation, without excessive blood loss eight patients in group 1 received blood transfusion, on the average 0.65 ± 0.51 liter of whole blood whereas during the remaining period at Huddinge Hospital, no more blood was given. Hb usually decreased during the first week of TPN probably due to rehydration by the TPN given. During the following period of preoperative TPN, Hb slowly increased, but the increase was not statistically significant (Table IV). In three patients, the prednisolone therapy was discontinued during or shortly after TPN. In two patients, where prednisolone was introduced during TPN, the corticosteroid medication was discontinued 10 weeks after TPN.

**Table IV:** Progress on preoperative total parenteral nutrition (TPN); selected blood (B-), serum (S-), and plasma (P-) concentrations (group 1).

	Before TPN			At the end of TPN			
			
Component	Mean	SD	*n*	Mean	SD	*n*	Reference range
Body weight (kg)	54.3	9.4	14	56.7**	8.3	14	
Body mass index (units)	18.6	1.8	14	19.4**	1.6	14	
B-hemoglobin (g/l)	107	12	13	112	10	14	115–165
Reticulocytes (%)	0.6	0.4	7	0.9	0.6	10	0.2–1.0
White cell count (10^9^/l)	10.1	3.6	11	5.7*	1.8	12	3.0–9.0
S-albumin (g/l)	34	6	13	39**	4	14	38–55
P-orosomucoid (g/l)	1.5	0.5	6	1.1	0.4	7	0.4–0.8
P-antitrypsin (g/l)	3.3	2.0	9	1.9	0.3	11	0.8–1.7
S-haptoglobin (g/l)	3.4	1.2	10	2.3**	0.9	13	0.4–2.5
S-ASAT (μkat/l)	0.35	0.33	13	0.47	0.43	14	0–0.70
S-ALAT (μkat/l)	0.37	0.74	13	0.62	0.99	14	0–0.70
S-ALP (μkat/l)	3.62	2.29	12	4.70	3.57	13	0–4.20
S-bilirubin, total (μmol/l)	7	3	11	9	4	13	0–26
S-cholesterol (mmol/l)	3.4	1.3	11	5.3**	1.2	12	2.6–6.4
S-triglycerides (mmol/l)	1.2	0.4	10	1.0*	0.4	12	0–2.2
S-free fatty acids (mmol/l)	0.70	0.26	5	0.95	0.36	7	0.15–0.92
S-triiodothyronine (nmol/l)	1.5	0.5	5	2.0*	0.5	8	1.2–2.3
S-thyroxine (nmol/l)	125	28	4	106	18	8	58–167
S-thyrotropic hormone (units/l)	2	1	4	3	2	5	0–6
S-immunoglobulin G (g/l)	11	3	10	13	4	13	7–17
S-immunoglobulin A (g/l)	3.0	1.7	10	3.1*	1.8	12	0.6–5.0
S-immunoglobulin M (g/l)	1.7	0.8	10	2.6**	1.0	12	0.7–3.7
S-phospholipids, total (mmol/l)	2.42^a^	0.52	7	3.63*^b^	0.20	5	2.41–3.05^c^
S-cephalin (mmol/l)	0.07^a^	0.02	7	0.12^b^	0.03	5	0.07–0.10^c^
S-lecithin (mmol/l)	1.66^a^	0.34	7	2.60*^b^	0.21	5	1.46–2.00^c^
S-lysolecithin (mmol/l)	0.17^a^	0.07	7	0.27^b^	0.04	5	0.17–0.32^c^
S-sphingomyelin (mmol/l)	0.53^a^	0.12	7	0.64^b^	0.05	5	0.43–0.57^c^

a In two cases measured on days 20 and 28, respectively, before TPN. Their states of malnutrition did not change clinically until TPN was instituted;

b Measured on days 46–57 of TPN;

c Wagener et al. [41].

* Significant difference from the value before TPN (*p* < 0.05);

** Significant difference from the value before TPN (*p* < 0.01).

The progress on the preoperative TPN given group 1 is summarized in Table IV. During the relevant period of TPN, the body weight (BW) and the body mass index (BMI) increased, as did also the serum concentrations of albumin, cholesterol, T_3_, IgA, and IgM, whereas the WBC, S-haptoglobin, and S-TG showed a decrease (Table IV). There was no metabolic complication such as hyperglycemia or need for insulin.

The liver function tests, S-ASAT, S-ALAT, and S-ALP, displayed no significant change, although S-ALP attained a level slightly above the normal range at the end of the preoperative period of TPN. The serum concentrations of both total and individual phospholipids showed increases during the TPN, and after 1.5 months of TPN, total S-phospholipids, S-lecithin, and S-sphingomyelin had attained levels above the corresponding reference levels.

## Discussion

The use of TPN as adjunct therapy for CD is currently used to improve nutritional status for surgery, but TPN only has not been found to be an alternative to resection [21]. Rombeau et al. [22] have found fewer surgical complications and lower mortality in a TPN-treated group compared with a control group, and Lashner et al. [10] reported that preoperative TPN was associated with reduced length of small bowel resection at the expense of longer hospital stay in bowel resection in CD.

Central venous catheterization is a well-established procedure, but there is a risk of complications on insertion of a CVC by percutaneous subclavian catheterization [23]. Although there were no severe lasting consequences related to the preoperative TPN, since all patients studied were in good condition when the intestinal resection was performed, it may be worth trying to shorten the preoperative TPN period from the mean of 46 days in this study to decrease the need of more than one CVC. The long average preoperative TPN period depended on that in the first patients, the TPN given was initially intended to be the sole therapy, but as intestinal resection could not be avoided, the goal for the preoperative TPN was shortened to 3 weeks with prolongation if necessary until acceptable remission had been achieved.

The control population was consecutively selected from all CD patients operated in Stockholm County during a preceding 20-year period, which probably will mean that some of them were not seriously ill at resection, whereas the 15 patients studied in a referral center were selected because they had a severe period of CD and intestinal resection could not be avoided. Although not calculated, it is therefore likely that the average CDAI of the control population was lower than the mean of the 15 TPN patients studied, as the controls included all patients and not only those with nutritional deficits and signs of inflammatory activity. The groups were not contemporaneous, but since there was no difference between them in preoperative treatment before the TPN given to group 1, they should be able to be compared.

In a study of 84730 patients who had undergone inpatient general and vascular surgery from 2005 through 2007 in five categories of hospitals in the USA, a rate of overall postoperative complications between 24.6% and 26.9% was found, whereas the rate of major complications varied between 16.2% and 18.2% [24]. After colonectomy, the rate of all postoperative complications varied between 24.7% and 28.1% [24], which is in accordance with the overall postoperative complication rate in group 2 (27.6%) in the present study.

During the period of preoperative TPN, the nutritional state improved as reflected in increases in BW, BMI, S-albumin [25,26], S-T_3_, and S-cholesterol, and the inflammatory activity in the intestines diminished as reflected in the decreases in WBC and S-haptoglobin. The changes in B-hemoglobin, P-orosomucoid, P-antitrypsin, and S-IgA pointed in the same advantageous directions, although they did not achieve statistical significance. Several mechanisms that are not mutually exclusive have been proposed to explain the apparent beneficial action of TPN in active CD [27].

A weakness of studies of TPN therapy for CD has been the failure to include controls to account for the natural history of the disease and the large placebo effect associated with the treatment of the disease [28]. Although prospective randomized controlled trials have become the accepted scientific standard for evaluating therapeutic efficacy, clinical nutrition research is not well suited for this methodology [29].

In this study, it is not possible to determine which of the variables studied is most important to improve during the TPN to minimize the risk of postoperative complications, although the increase in S-albumin may be crucial [30], since the S-albumin concentration may reflect both the nutritional state and the degree of inflammatory activity in CD [26]. If there would be no increase in S-albumin after 3 weeks of TPN, the TPN may be prolonged if it is possible, until an increase is obtained, since absence of increase in S-albumin may be associated with increased risk of postoperative complications [25]. Moreover, the risk of developing postoperative complications has been found to be inversely related to the concentration of S-albumin [30]. Also the absence of respiratory complications found may be explained by the improved nutritional state during the preoperative TPN [31].

The minimum effective dose of preoperative TPN could not be determined accurately in the study presented. Lashner et al. [10] surmised that preoperative TPN for more than 10 days in CD had little confirmed beneficial effect in their sample, but their TPN patients started on S-albumin levels around 35–37 g/l and may thus have had somewhat less nutritional deficits and less inflammation than the patients in group 1 whose initial level was around 34 g/l.

The increase in BW during the preoperative TPN given may also have contributed to a decreased risk of postoperative complications, since recent preoperative weight loss is a basic indicator of surgical risk [32].

The increase in S-T_3_ within the normal range, while S-T_4_ showed no significant change in this study, may reflect a normalization of the resting metabolic rate (RMR) from a state of relative starvation or underfeeding with lowered RMR [33]. Thyroid hormones carry a major responsibility for regulating the RMR. T_3_ production is decreased in starvation with little change in T_4_ production rates [34]. The concentrations of total, free and production rates of the ther-mogenic hormone, T_3_, are increased by overfeeding and decreased by underfeeding in concert with the changes in RMR that are found in these conditions [33]. Since there is a strong correlation between the changes in total T_3_ concentrations and the relative amount kcal/24 h/kg fat free mass [33], the increase in S-T_3_ found probably reflects an improvement in the RMR in the lean patients studied.

Despite no adverse clinical effect was observed such as liver function disturbance, the increases above reference values in the serum phospholipids during the TPN studied are remarkable, but may be explained by the fact that by the 500 ml Intralipid 10% given daily, 12 g of egg-yolk phospholipids were provided per 24 h, an amount that may be unnecessary high. However, the manufacturing pharmaceutical company has, after this study, diminished the content of phospholipids to 6 g in 500 ml Intralipid 10%.

Since there is an association between blood transfusion and increased rates of postoperative bacterial infections [35,36], and only one patient was given blood transfusion during the preoperative TPN studied and seven patients received no blood at operation, this restraint with blood transfusion may also have contributed to the absence of postoperative complications.

It is desirable to be able to diminish or discontinue prednisolone administration during TPN, because the protein catabolic rate will increase if the prednisolone dose is increased, and there is an increased risk of postoperative as well as infectious complications following the preoperative use of corticosteroids in patients with CD [37], although Bruewer et al. [38] found no increased postoperative complication rate. Thus, when the signs of clinical remission were unsatisfactory during steroid-free TPN, there showed to be no drawback to include prednisolone to induce a satisfactory preoperative remission in the two patients who were clinically judged to need this drug [39]; both had an uneventful postoperative recovery and were off the prednisolone medication 10 weeks later.

Consequently, this study shows that preoperative TPN for at least 18 days may be recommended to be given to patients with moderate to severe CD until clinical remission is achieved in order to minimize the risk of early postoperative complications after intestinal resection and primary anastomosis.
